# Supplementary material for: Investigating the etiologies of non-malarial febrile illness in Senegal using metagenomic sequencing
Source: Nat Commun. 2024 Jan 25;15:747. doi: 10.1038/s41467-024-44800-7 (PMC10810818; doi:10.1038/s41467-024-44800-7)
Supplement: Supplementary file 1 — Supplementary Information [file 41467_2024_44800_MOESM1_ESM.pdf]

a

		2018				2019			
		**Febrile**		**Healthy**		**Febrile**		**Healthy**	
Female	*Adult (18+)*	79	(24.6%)	151	(38.8%)	51	(25.0%)	32	(30.8%)
	Adolescent (13-17)	26	(8.1%)	31	(8.0%)	14	(6.9%)	6	(5.8%)
	Child (6-12)	26	(8.1%)	42	(10.8%)	20	(9.8%)	13	(12.5%)
	Young child (2-6)	8	(2.5%)	15	(3.9%)	8	(3.9%)	6	(5.8%)
Male	*Adult (18+)*	97	(30.2%)	45	(11.6%)	59	(28.9%)	21	(20.2%)
	Adolescent (13-17)	22	(6.9%)	33	(8.5%)	14	(6.9%)	10	(9.6%)
	Child (6-12)	42	(13.1%)	48	(12.3%)	31	(15.2%)	8	(7.7%)
	Young child (2-6)	21	(6.5%)	24	(6.2%)	7	(3.4%)	8	(7.7%)
	Total	**321**		**389**		**204**		**104**	

b

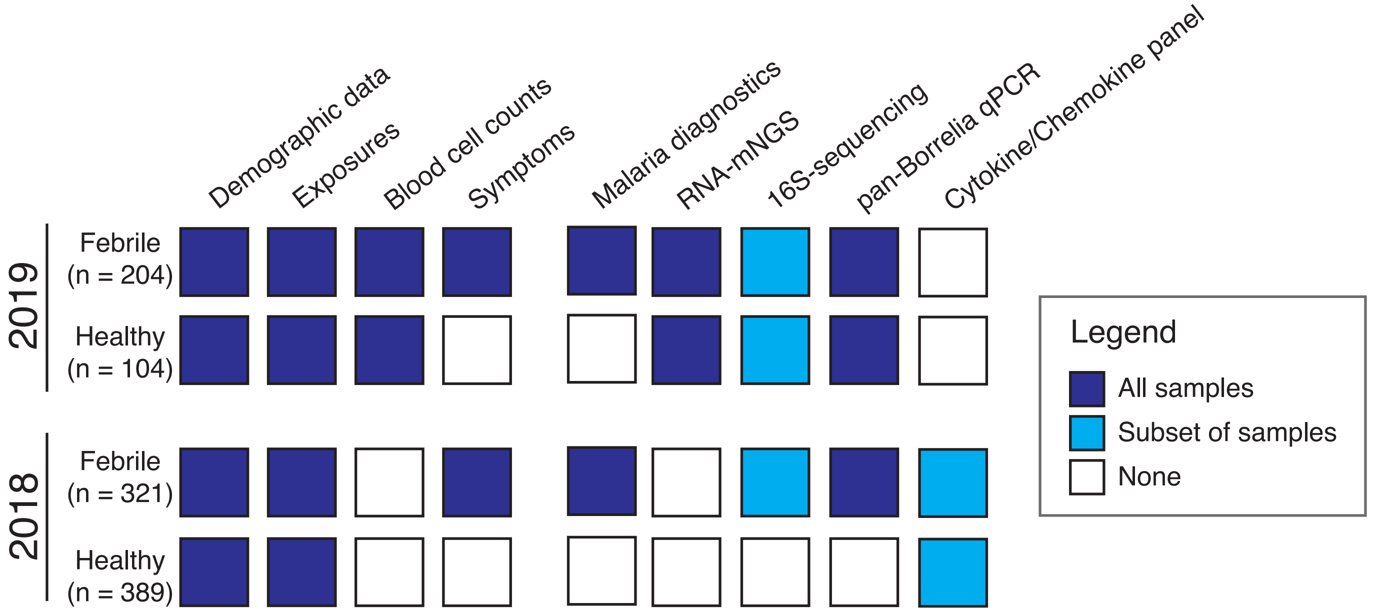

Supplementary Figure 1: Overview of data set in the study **a.** Demographics of febrile patients and healthy controls enrolled in the study from 2018-2019. **b.** Summary of metadata, clinical diagnostics, and lab data available for febrile cases and healthy controls in 2018 and 2019.

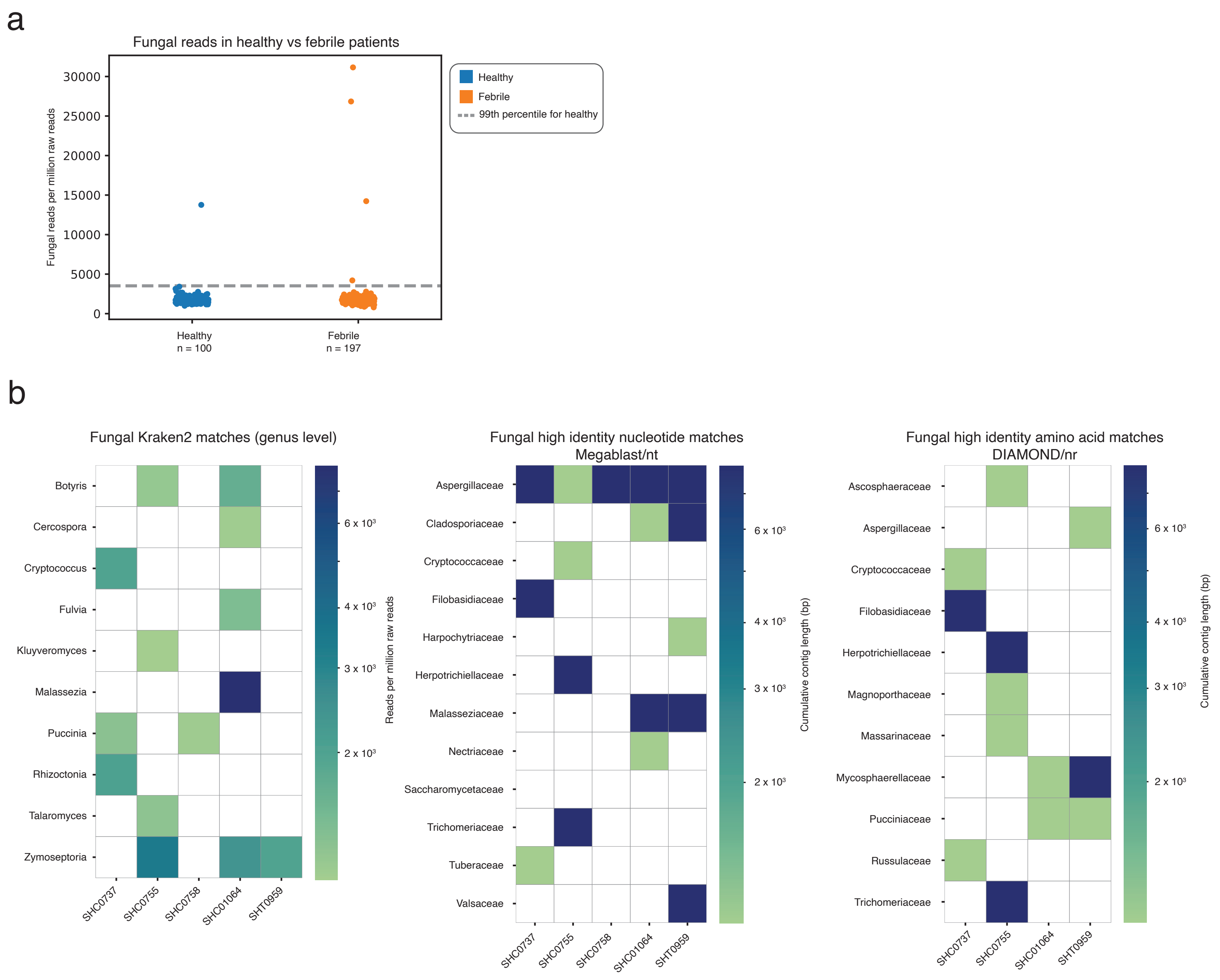

Supplementary Figure 2: a. Proportion of reads classified as fungal (reads per million raw reads, Kraken2) in febrile cases and healthy controls. **b.** Taxonomic classifaction (Kraken2) of fungal reads; color indicates reads per million, filtered to show only samples/genera with >1000 rpm. **c.** Fungal de novo contigs (SPAdes) classified by nucleotide search (megablast / nt) with > 90% sequence identity and >30% query coverage, filtered to show only samples/families with cumulative contig length > 1kb. **d.** Fungal de novo contigs (SPAdes) classified by translated nucleic acid search (DIAMOND-blastx / nr) with > 90% sequence identity and >30% query coverage, filtered to show only samples/families with cumulative contig length > 1kb. Source data are provided as a Source Data file

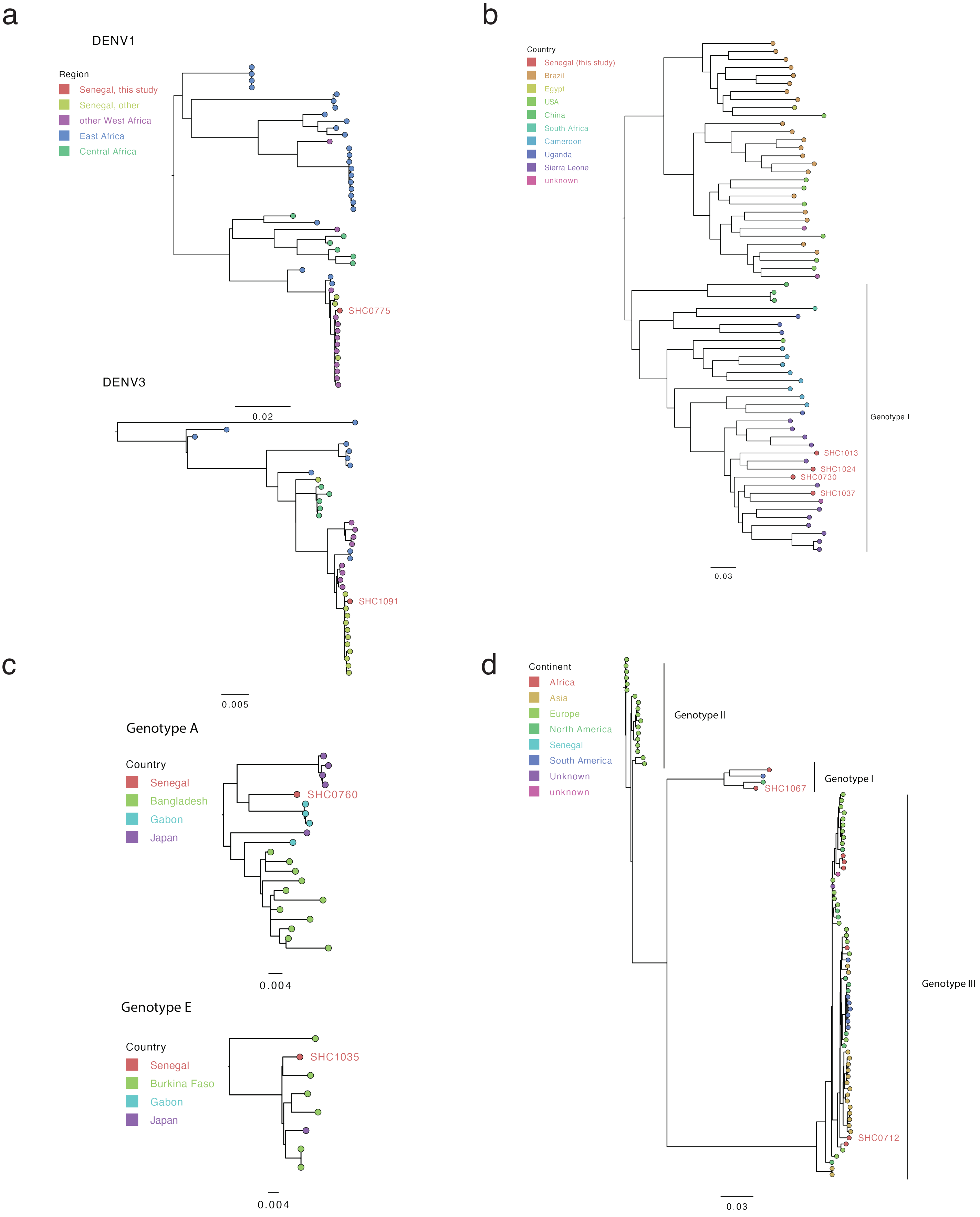

Supplementary Figure 3: Viral isolate phylogenetic trees **a.** Maximum likelihood phylogenetic tree (IQ-TREE) of DENV1 and DENV3 genomes from this study (red) in the context of all > 80% complete genomes from Africa in NCBI virus. **b.** Maximum likelihood phylogenetic tree (IQ-TREE) Human Pegivirus 1 genomes from this study (red) in the context of all >80% complete genomes colored by country of origin. **c.** Maximum likelihood phylogenetic tree (IQ-TREE) of Hepatitis B genomes from this study (red) in the context of all >80% complete Genotype A or Genotype E Hepatitis B virus genomes from Africa in NCBI virus. **d.** Maximum likelihood phylogenetic tree (IQ-TREE) for Parvovirus B19 genomes from this study (red) in the context of all >80% complete Parvovirus B19 genomes from a human host in NCBI virus.

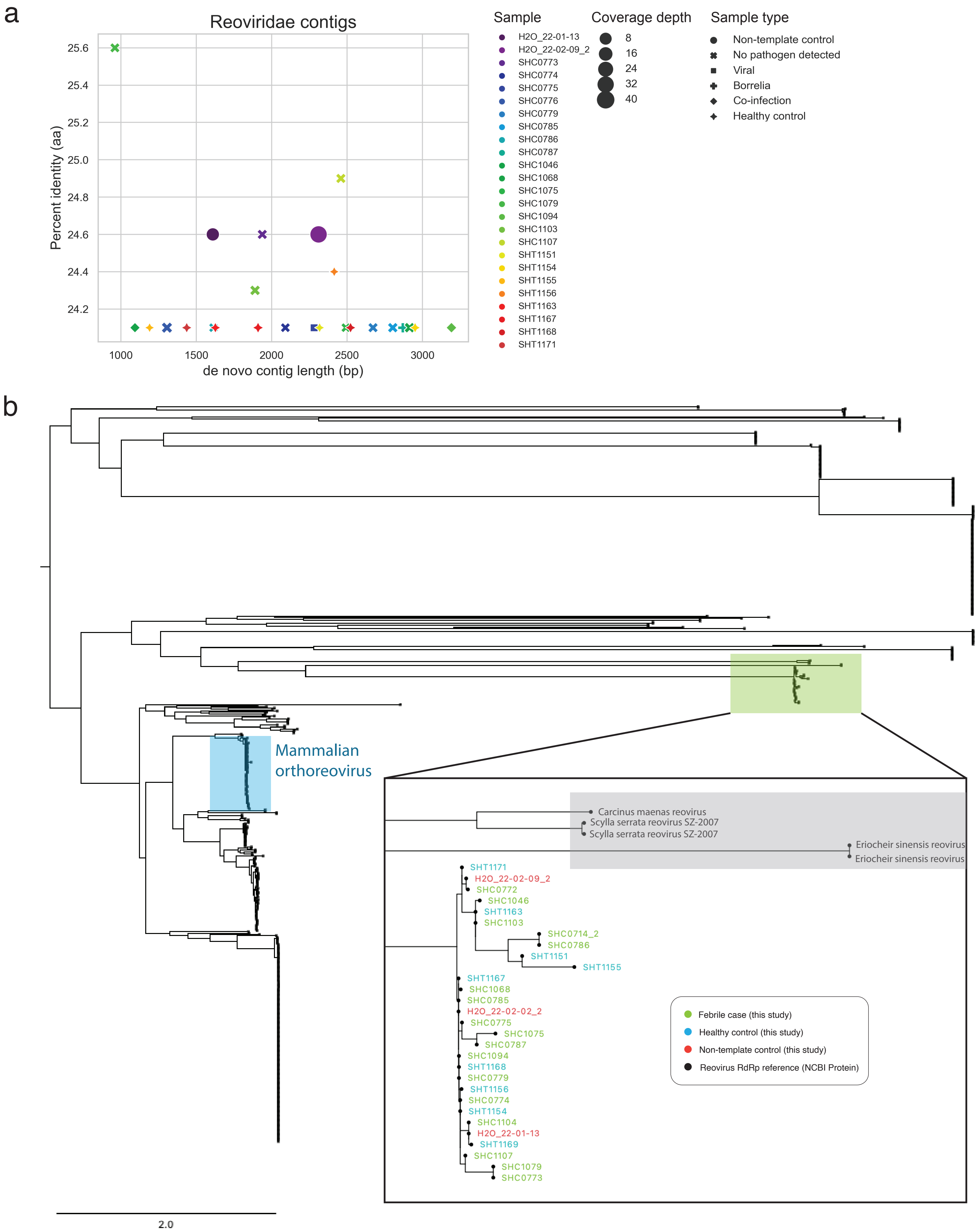

Supplementary Figure 4: Divergent *Reoviridae de novo* contigs identified in RNA-mNGS with **a.** percent amino acid identity to the closes DIAMOND-blastx match on the y-axis and contig length on the x-axis. Each dot represents a contig, colored by the sample ID with shape indicating the type of sample and size of the marker indicating the mean read depth across the contig. **b.** Maximum likelihood phylogenetic tree of available RdRp amino acid sequences for *Reoviridae* and *de novo* contigs from febrile patients (green), healthy controls (blue) and non-template controls (red) from this cohort. Source data are provided as a Source Data file.

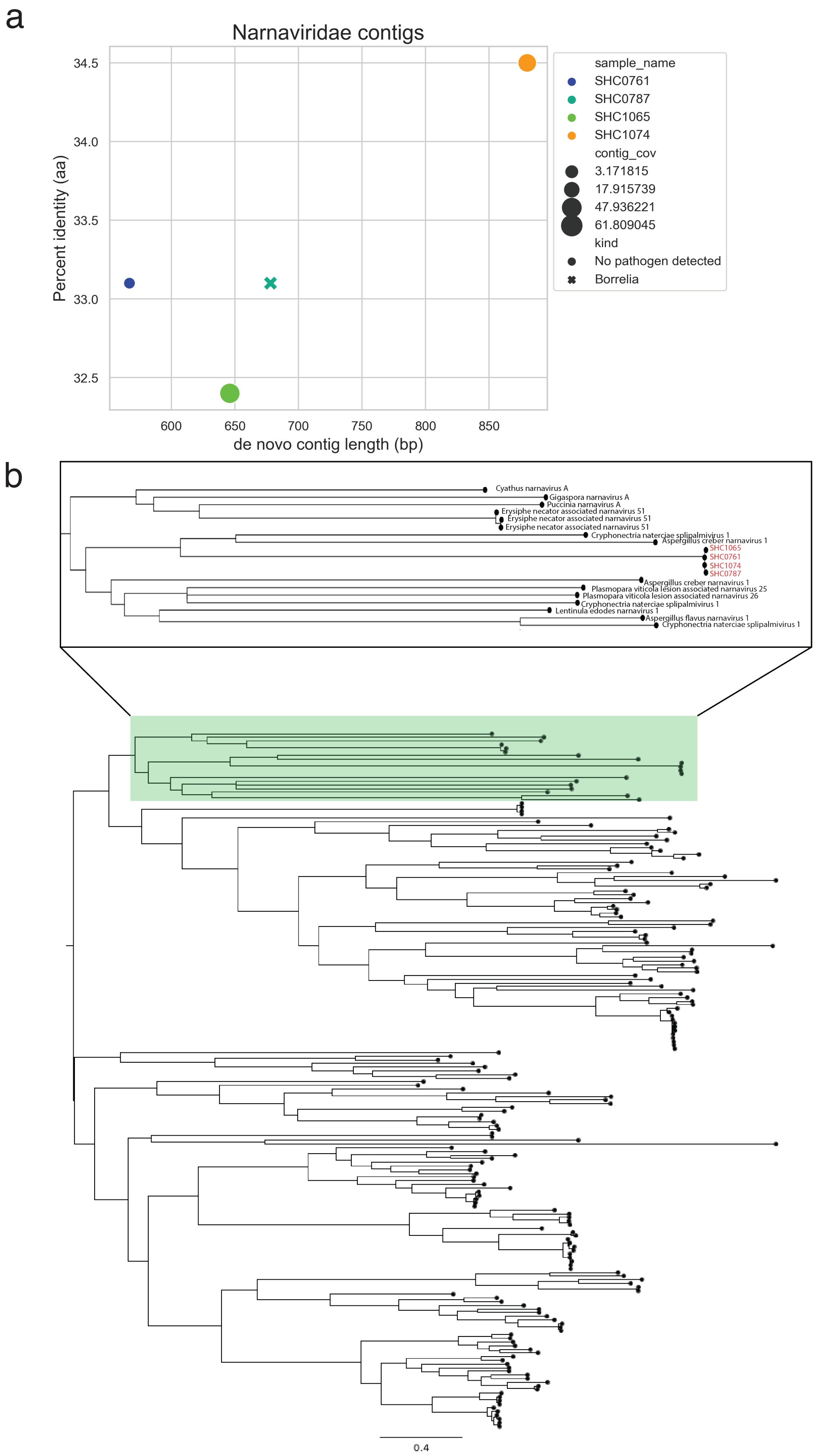

Supplementary Figure 5: Divergent *Narnaviridae de novo* contigs identified in this cohort with **a.** percent amino acid identity to the closes DIAMOND-blastx match on the y-axis and contig length on the x-axis. Each dot represents a contig, colored by the sample ID with shape indicating sample type and the size of the marker indicating the mean read depth across the contig. **b.** Maximum likelihood phylogenetic tree of available RdRp amino acid sequences for *Narnaviridae* and *de novo* contigs from febrile patients in this cohort (red). Source data are provided as a Source Data file.

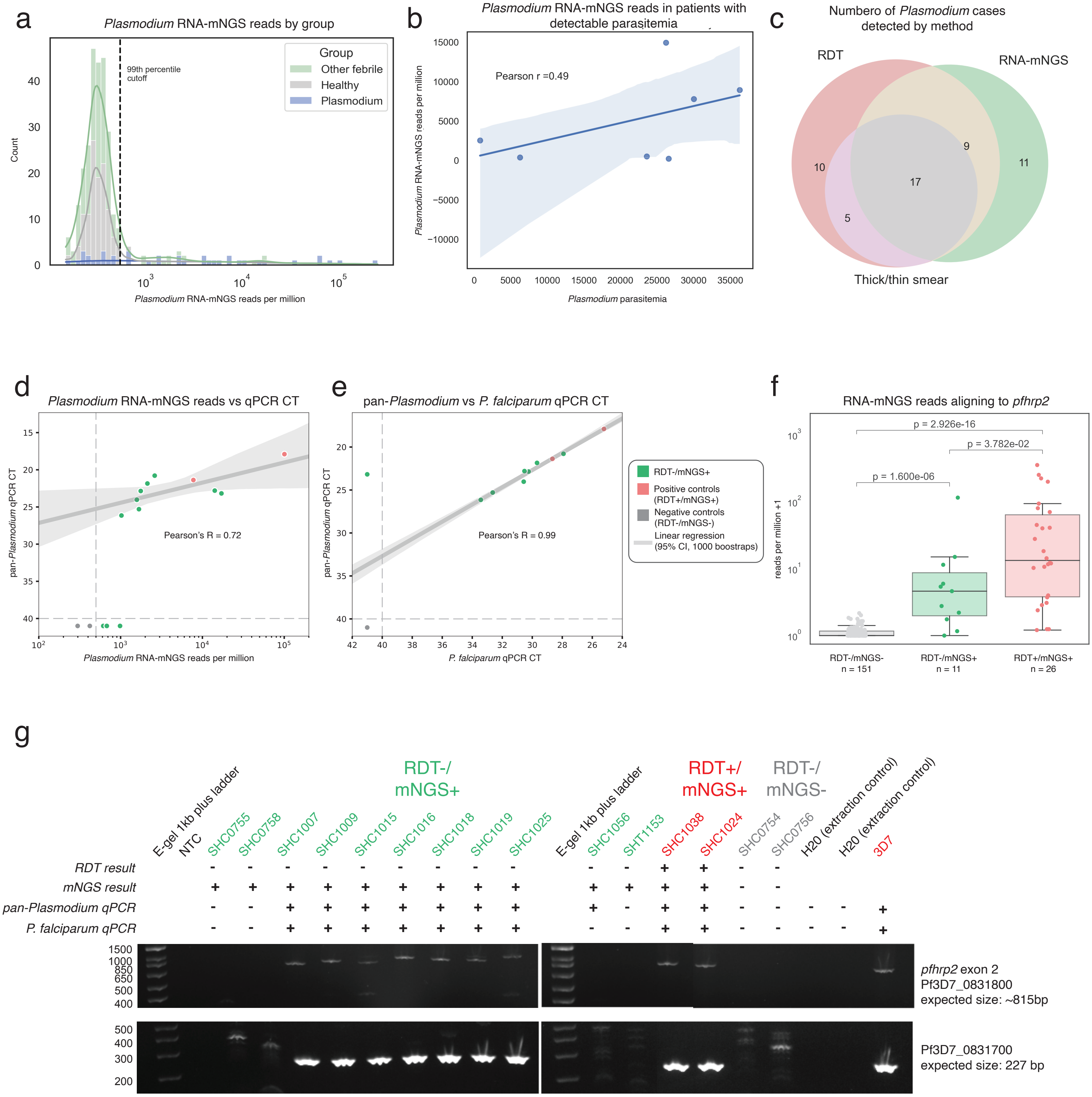

Supplementary Figure 6: a. Distribution of RNA-mNGS read proportion in healthy, *Plasmodium* RDT(+) febrile and *Plasmodium* RDT(-) febrile patients with 99th percentile of *Plasmodium* abundance in healthy individuals, the threshold for considering sample *Plasmodium* positive by RNA-mNGS, marked by dashed line. **b.** *Plasmodium* RNA-mNGS reads per million raw reads vs parasite density for thick/thin blood smear positive patients with detectable parasitemia. **c.** *Plasmodium* detection by rapid diagnostic test (RDT), RNA-mNGS, or thick/thin smear. **d.** *Plasmodium* RNA-mNGS reads per million (plasma) vs pan-*Plasmodium* CT (dried blood spot) and **e.** *pan-Plasmodium* CT vs *P. falciparum* CT. Dashed lines represent the cutoffs for considering a sample positive. For qPCR assays, samples were considered positive if CT was < 40 in 3/3 technical replicates and mean CT across replicates is plotted for each sample. For negative samples, CT was set to 41 for visualization. Regressions were performed only on positive samples. **f.** Reads per million raw reads aligned to *pfhrp2* (PlasmoDB PF3D7_0831800), the gene encoding PfHRP-2, the target antigen target for the *P. falciparum* RDTs. Box plots show mean and interquartile range; whiskers extend to points within 1.5 IQRs of the lower and upper quartile. **g.** Amplification of *pfhrp2* exon 2 and the flanking gene Pf3D7_0831700, visualized on E-gel EX 2% with E-gel 1kb plus ladder. RDT+/mNGS+ samples and 3D7, a *P. falciparum* line with intact *pfhrp2,* are shown for comparison. Source data are provided as a Source Data file.

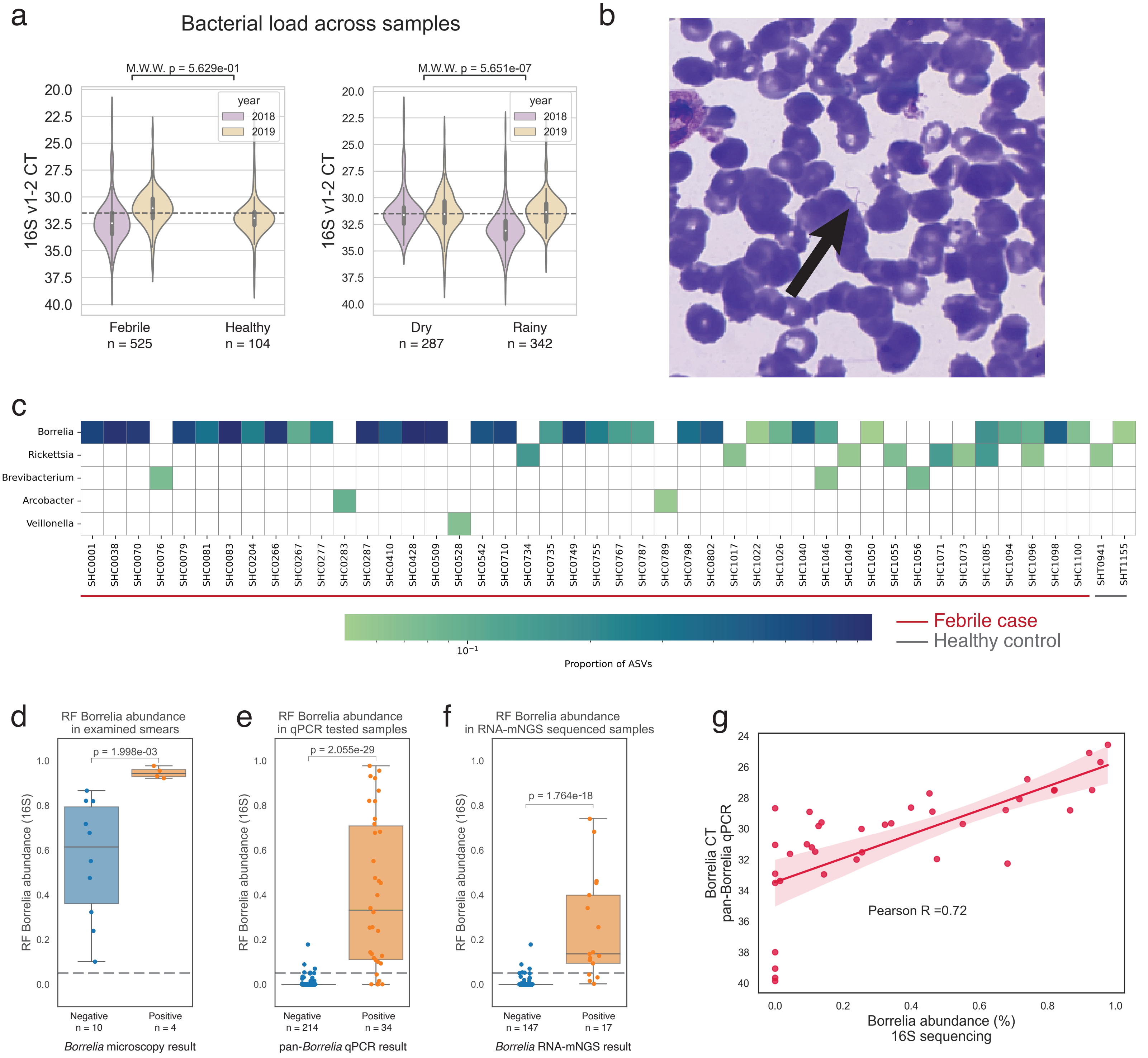

h

Borrelia 16S v1-2 rRNA abundance

		≥ 5% (+)	< 5% (-)
pan-*Borrelia* qPCR	Positive	28	6
	Negative	6	208

Sensitivity: 0.82
Specificity: 0.97
PPV: 0.82
NPV: 0.97

i

Borrelia RNA-mNGS abundance

		≥ 1 rpm (+)	< 1 rpm (-)
pan-*Borrelia* qPCR	Positive	17	0
	Negative	7	283

Sensitivity: 1.0
Specificity: 0.98
PPV: 0.71
NPV: 1.0

Supplementary Figure 7: a. Bacterial load across samples measured by eubacterial V1-2 qPCR; violin plot shows the kernel density estimate and quartile ranges with whiskers. Dashed line indicates the 31.5 CT cutoff used to select samples for 16S sequencing. **b.** Representative Giemsa stained blood smear (100X magnification) from a *Borrelia* smear positive patient with arrow indicating the spirochete. **c.** Bacterial pathogens identified by 16S across both years in febrile cases (red line) and healthy controls (grey line). *Borrelia* abundance by 16S (ASVs classified as *Borrelia* at the genus level / total ASVs in sample) for samples positive and negative for *Borrelia* by **d.** blood smear examination, **e.** pan-*Borrelia* qPCR and **f.** RNA-mNGS. P-values represent the result of Mann-Whitney-Wilcoxon test, two-sided. Box plots show mean and interquartile range; whiskers extend to points within 1.5 IQRs of the lower and upper quartile. **g.** Quantification of *Borrelia* load by pan-*Borrelia* qPCR (y-axis) vs v1-2 ASV abundance (x-axis). Shading represents the 95% confidence interval for the linear regression with 1000 bootstraps. **h.** Sensitivity and specificity of 16S sequencing and **i.** RNA-mNGS sequencing, compared to pan-*Borrelia* qPCR. Source data are provided as a Source Data file.

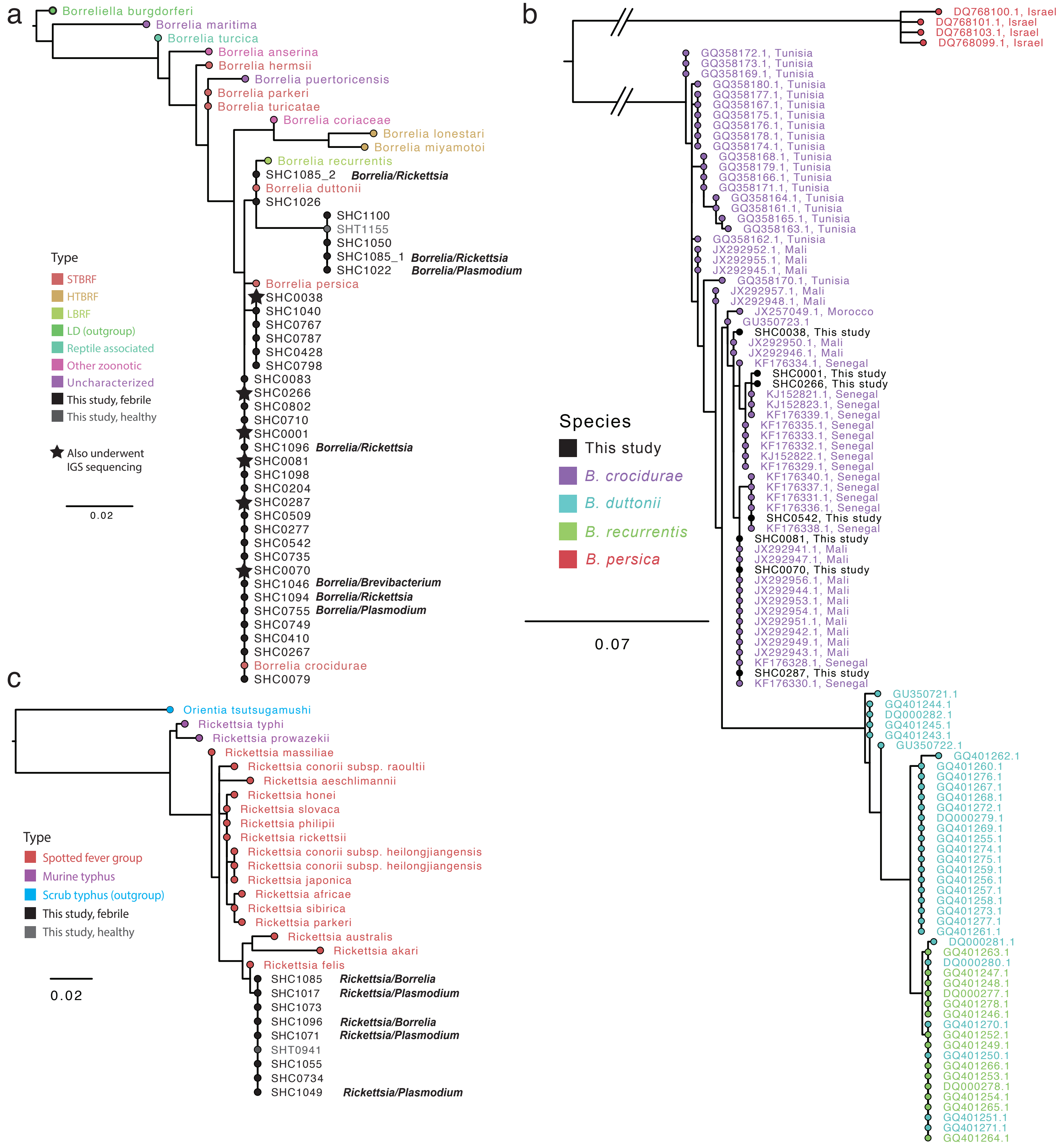

Supplementary Figure 8: Maximum likelihood phylogenetic trees (IQ-TREE) of **a.** 16S v1-2 rRNA gene sequences for *Borrelia* from this study (febrile: black, healthy: grey) in the context of relapsing fever *Borrelia* sequences from the curated NCBI 16S rRNA target loci project and Silva non-redundant reference (Silva Ref NR) small subunit database (1 sequence per species) rooted on the outgroup, *Borreliella burgdorferi*. Stars indicate samples that also underwent IGS sequencing. **b.** IGS sequences from this study (black) in the context of available reference sequences for *B. crocidurae* (purple), *B. duttonii* (cyan), *B. recurrentis* (green), and *B. persica* (red), midpoint rooted and **c.** 16S v1-2 rRNA gene sequences for *Rickettsia* from this study (febrile: black, healthy: grey) in the context of *Rickettsia* sequences from the curated NCBI 16S rRNA target loci project Silva Ref NR small subunit database (1 sequence per species, species associated with human disease only) rooted on the outgroup, *Orientia tsutsugamushi*. In the case of multiple v1-2 sequences for a given taxa in a given sample, sequences are distinguished by "_1" and "_2". In the case of co-infections, italic text next to the sample name lists the co-infecting taxa.

a

Model type:<br>*Comparison group(s):*<br>*Training set:*<br>*Testing set:*	**Clinical only**<br>**All febrile**<br>**2018-2019 (n = 526)**<br>**2018-2019 (n = 526)**	**Clinical only**<br>**Other NMFI**<br>**2018 (n = 288)**<br>**2019 (n = 163)**	**Clinical only**<br>**Other NMFI**<br>**2019 (n = 163)**<br>**2019 (n = 163)**	**Clinical + CBC**<br>**Other NMFI**<br>**2019 (n = 163)**<br>**2019 (n = 163)**
**Recall (95% CI)**	0.922 (0.909-0.944)	0.832 (0.797-0.889)	0.815 (0.710-0.885)	0.853 (0.769 – 0.909)
**Precision (95% CI)**	0.847 (0.806-0.893)	0.776 (0.730-0.878)	0.773 (0.625 – 0.833)	0.799 (0.767-0.833)
**F1 (95% CI)**	0.883 (0.857-0.902)	0.802 (0.774-0.854)	0.793 (0.678-0.853)	0.825 (0.769 – 0.870)
**AUC/ROC (95% CI)**	0.921 (0.911-0.937)	0.864 (0.821-0.920)	0.871 (0.808-0.934)	0.867 (0.842-0.895)
**AUC/PR (95% CI)**	0.918 (0.888-0.956)	0.843 (0.757-0.931)	0.871 (0.819-0.923)	0.828 (0.760-0.898)

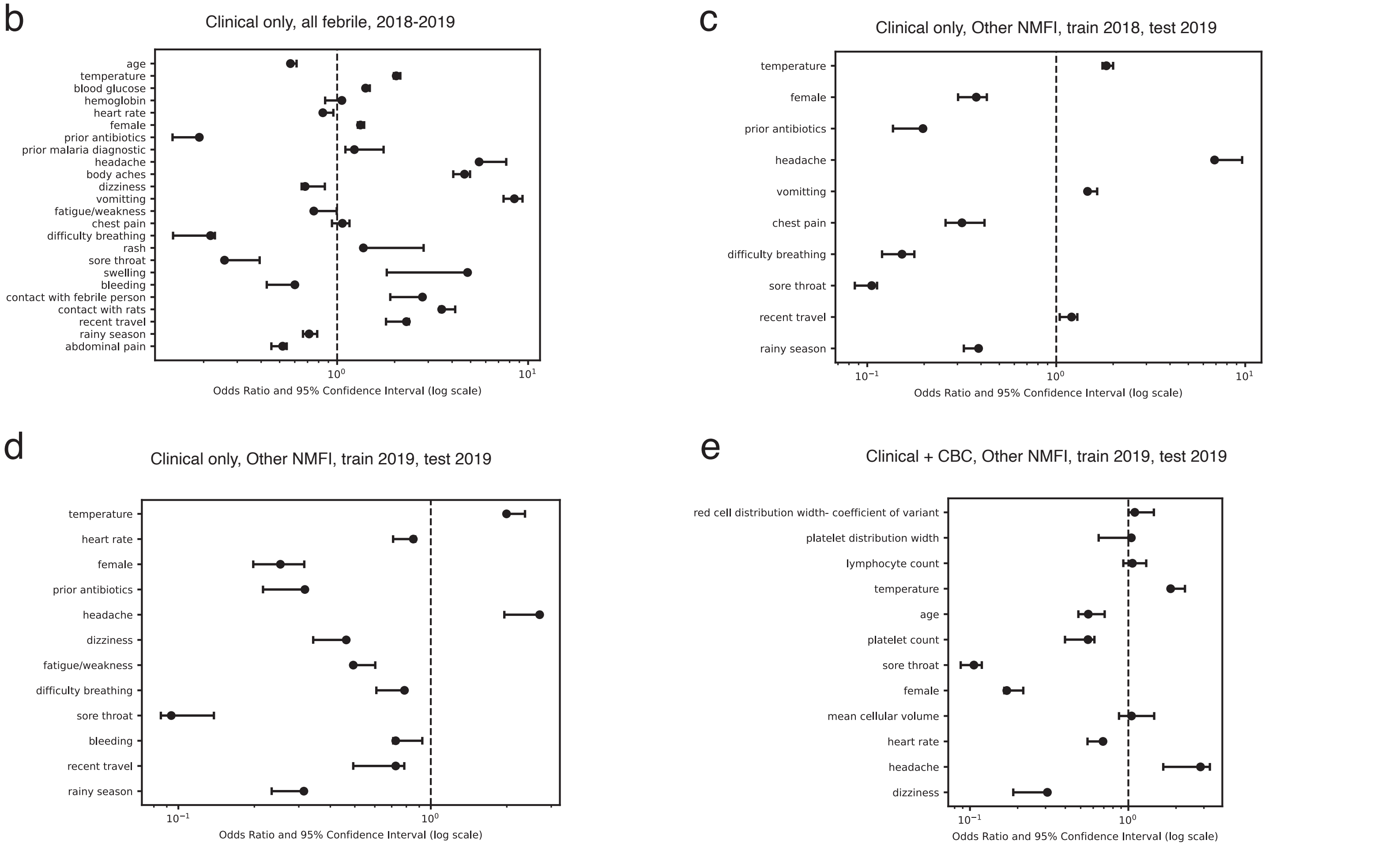

Supplementary Figure 9: a. Performance (tested with bootstrapping with 5-fold cross validation) of weighted logisistic regression models to distinguish *Borrelia* infection from all other febrile illness (All febrile) or all non-malarial febrile illness (Other NMFI) using clinical data, including demographics, symptoms, exposures, and vital signs (Clinical only) or clinical data and complete blood counts with differential (Clinical + CBC). Mean odds ratios (center point) with 95% confidence interval (error bars) shown for model features in **b.** the clinical only model to distinguish *Borrelia* from all other febrile, trained and tested on the full dataset from 2018-2019 (n = 526), **c.** the clinical only model to distinguish *Borrelia* from other NMFI, trained on the 2018 dataset (n = 288) and tested on the 2019 dataset (n = 163), **d.** the clinical only model to distuinguish *Borrelia* from other NMFI trained and tested on the 2019 dataset (n = 163), and **e.** the clinical + CBC model to distuinguish *Borrelia* from other NMFI, trained and tested on the 2019 dataset (n = 163). Source data are provided as a Source Data file.

Supplementary Table 1: Bacterial pathogens in febrile vs healthy patients and *P. falciparum* RDT positives vs RDT negative patients

		Febrile vs healthy			*P. falciparum* RDT+ vs RDT- (febrile patients only)		
Pathogen	*Detection Method*	*Febrile samples positive*	*Healthy samples positive*	*p-value (Fisher exact, two-sided)*	*RDT+ samples positive*	*RDT- samples positive*	*p-value (Fisher exact, two-sided)*
Borrelia	qPCR	7.4% (39/526)	0% (0/104)	0.0012	0% (0/75)	8.7% (39/448)	0.0033
Borrelia	16S sequencing	15.5% (33/213)	2.9% (1/35)	0.0593	2.8% (1/36)	18.2% (32/176)	0.0209
Rickettsia	16S sequencing	3.8% (8/213)	2.9% (1/35)	1.0000	5.6% (2/36)	3.4% (6/176)	0.6260

Supplementary Table 2: Viral pathogens detected in 2019

Virus	Genotype	Season	Patient age (years)	Biosample Accession	Nucleotide Accession
Dengue Virus 1	Related to but not part of Genotype III	Dry Season	22	SAMN34409179	OR800007
Dengue Virus 3	Genotype III	Wet Season	29	SAMN34409290	OR800009
Hepatitis B Virus	Genotype A	Dry Season	10	SAMN34409164	OR800013
Hepatitis B Virus	Genotype E	Wet Season	12	SAMN34409235	OR800011
Parvovirus B19	Genotype III	Dry Season	7	SAMN34409120	OR800006
Parvovirus B19	Genotype I	Wet Season	37	SAMN34409267	OR800008
HIV-1		Dry Season	24	SAMN34409124	
Pegivirus C	Genotype I	Dry Season	48	SAMN34409136	OR800014
Pegivirus C	Genotype I	Wet Season	10	SAMN34409214	OR800012
Pegivirus C	Genotype I	Wet Season	33	SAMN34409225	
Pegivirus C	Genotype I	Wet Season	26	SAMN34409237	OR800010

Supplementary Table 3: Investigation of RDT negative *Plasmodium* infections

Sample	Case or control?	Age group	Season	Plasmodium RNA-mNGS reads	Prior RDT?	Number of previous RDTs	Prior antimalairals?	pan-*Plasmodium* qPCR result#	*P. falciparum* qPCR result#
SHC0755	Febrile	Adult	Dry	6.1E+02	No		No	FALSE	FALSE
SHC0758	Febrile	Young child	Dry	9.8E+02	No		No	FALSE	FALSE
SHC1007	Febrile	Child	Rainy	1.8E+03	No		No	TRUE	TRUE
SHC1009	Febrile	Adult	Rainy	1.6E+03	No		No	TRUE	TRUE
SHC1015	Febrile	Young child	Rainy	1.0E+03	No		No	TRUE	TRUE
SHC1016	Febrile	Adult	Rainy	2.6E+03	No		No	TRUE	TRUE
SHC1018	Febrile	Child	Rainy	2.1E+03	No		No	TRUE	TRUE
SHC1019	Febrile	Adolescent	Rainy	1.7E+03	No		missing	TRUE	TRUE
SHC1025	Febrile	Adult	Rainy	1.4E+04	No		No	TRUE	TRUE
SHC1056	Febrile	Adult	Rainy	1.7E+04	Yes	5	No	TRUE	FALSE
SHT1153	Healthy	Adult	Rainy	6.8E+02	missing		missing	FALSE	FALSE

**Young child: 2-6, child: 6-12 years, adolescent: 13-17 years, adult 18+*
# CT < 40 in 3/3 technical replicates is a positive test

Supplementary Table 4: Primer sequences

Name	***Sequence***	***Assay***	***Reference***
V1/2-F	5'-CTGCTGCCTCCCGTAGGAGT-3'	Total bacterial load qPCR	Kingry, 2020
V1/2-R	5'-AGAGTTTGATCCTGGCTCAG-3'		
Borrelia-F	5'-AGCYTTTAAAGCTTCGCTTGTAG-3'	pan-Borrelia qPCR	Kingry, 2018
Borrelia-R	5'-GCCTCCCGTAGGAGTCTG-3'		
Spp-F	5'-AGCTCTTTCTTGATTTCTTGG-3'	pan-*Plasmodium* qPCR	Lazrek, 2023
Spp-R	5'-CAGACAAATCATATTCACGAACT-3'		
Fal-F	5'-CCGACTAGGTGTTGGATGAAAGTGTTAA-3'	*P. falciparum* qPCR	
Fal-R	5'-AACCCAAAGACTTTGATTTCTCATAA-3'		
Fal probe	/5Cy5/CT ATC TAA A/TAO/A GAA ACA CTC AT/3IAbRQSp/		
Tail-V1/2-F	5'-ACACTCTTTCCCTACACGACGCTCTT CCGATCTCTGCTGCCTCCCGTAGGAGT-3'	16S sequencing library construction	Kingry, 2020
Tail-V1/2-R	5'-GTGACTGGAGTTCAGACGTGTGCTCTT CCGATCTAGAGTTTGATCCTGGCTCAG-3'		
IGS-outer-F	5'-GTATGTTTAGTGAGGGGGGTG-3'	IGS sequencing	Bunikis, 2004
IGS-outer-R	5'-GGATCATAGCTCAGGTGGTGAG-3'		
IGS-inner-F	5'-AGGGGGGTGAAGTCGTAACAAG-3'		
IGS-inner-R	5'-GTCTGATAAACCTGAGGTCGGA-3'		
Pf3D7_0831800-F	5'-CAAAAGGACTTAATTTAAATAAGAG-3'	*pfhrp2* and flanking gene amplification	Cheng, 2014
Pf3D7_0831800-R	5'-AATAAATTTAATGGCGTAGGCA-3'		
Pf3D7_0831700-F	5'-AGACAAGCTACCAAAGATGCAGGTG-3'		
Pf3D7_0831700-R	5'-TAAATGTGTATCTCCTGAGGTAGC-3'		

Supplementary Table 5: Synthetic DNA standard sequences

Name	***Sequence***	***Assay***
B. duttonii v1-4	5'-<br>TTTACAGCGTAGACTACCAGGGTATCTAATCCTGTTTGCTCCCTACGCTTTCGTGACTC<br>AGCGTCAGTCTTGACCTAGAAGTTCGCCTTCGCCTCTGGTATTCTTCCTGATATCAACA<br>GATTCCACCCTTACACCAGGAATTCTAACTTCCCCTATCAGACTCTAGTCATGCAGTTTC<br>TAGCATAGCTCCACAGTTGAGCTGTGGTATTTTACGCACAGACTTGCATATCCGCCTAC<br>TCACCCTTTACGCCCAATAATCCCGAACAACGCTCGCCCCTTACGTATTACCGCGGCTG<br>CTGGCACGTAATTAGCCGGGGCTTATTCATAAATTAACGTCATCACCTTGTCATTTCCTA<br>CAAAGCTTATTCCTCATTTATAAAAGAACTTTACAATCTTTCGACCTTCTTCGTTCACGCA<br>GTGTCGCTCCGTCAGGCTTTCGCCCATTGCGGAAGATTCTTAGCTGCTGCCTCCCGTA<br>GGAGTCTGGACCGTATCTCAGTTCCAGTGTGACCGTTCACCCTCTCAGGCCGGTTACT<br>TATCATAGCCTTGGTAGGCTCTTACCCTACCAACTAGCTAATAAGACGCAGACTCATCT<br>ACAAGCGAAGCTTTAAAGGCTTCCTTTCATCAATTGACATCTCAACTGACCTTATTCGGT<br>ATTAGCTACTATTTCTAATAGTTATCCCCATCTCATAGGTAGATTATCCACGCGTTACTC<br>ACCCGTTCGCCACTGAATGTATTGCTACATCCCGTTTGACTTGCATGCTTAAGACGCAC<br>TGCCAGCGTTAGTTCTAAGCCAGGATCAAACTCTTCGTTATTTT-3'	Total bacterial load qPCR,<br>pan-Borrelia qPCR
Pf_PlassSpp_gblock	5'-<br>CGAAGTGCGTCTAGTCCTTGTATCCAAGGCAAAGCTCTTTCTTGATTTCTTGGATGGTG<br>ATGCATGGCCGTTTTTAGTTCGTGAATATGATTTGTCTGCCGACTAGGTGTTGGATGAA<br>AGTGTTAAAAATAAAAGTCATCTTTCGAGGTGACTTTTAGATTGCTTCCCTCAGTACCTT<br>ATGAGAAATCAAAGTCTTTGGGTTTAAGCAAGAGTCTGCGACTCCTGTGGATCTAC-3'	pan-*Plasmodium* qPCR,<br>*P. falciparum* qPCR
