# Supplementary material for: Investigating the etiologies of non-malarial febrile illness in Senegal using metagenomic sequencing
Source: Nat Commun. 2024 Jan 25;15:747. doi: 10.1038/s41467-024-44800-7 (PMC10810818; doi:10.1038/s41467-024-44800-7)
Supplement: Supplementary file 3 — Reporting Summary [file 41467_2024_44800_MOESM3_ESM.pdf]

nature portfolio

Corresponding author(s): Katherine J. Siddle

Last updated by author(s): Aug 24, 2023

Reporting Summary

Nature Portfolio wishes to improve the reproducibility of the work that we publish. This form provides structure for consistency and transparency in reporting. For further information on Nature Portfolio policies, see our Editorial Policies and the Editorial Policy Checklist.

Statistics

For all statistical analyses, confirm that the following items are present in the figure legend, table legend, main text, or Methods section.

n/a	Confirmed	
☐	☒	The exact sample size (*n*) for each experimental group/condition, given as a discrete number and unit of measurement
☒	☐	A statement on whether measurements were taken from distinct samples or whether the same sample was measured repeatedly
☐	☒	The statistical test(s) used AND whether they are one- or two-sided<br>*Only common tests should be described solely by name; describe more complex techniques in the Methods section.*
☒	☐	A description of all covariates tested
☐	☒	A description of any assumptions or corrections, such as tests of normality and adjustment for multiple comparisons
☐	☒	A full description of the statistical parameters including central tendency (e.g. means) or other basic estimates (e.g. regression coefficient) AND variation (e.g. standard deviation) or associated estimates of uncertainty (e.g. confidence intervals)
☐	☒	For null hypothesis testing, the test statistic (e.g. *F*, *t*, *r*) with confidence intervals, effect sizes, degrees of freedom and *P* value noted<br>*Give P values as exact values whenever suitable.*
☒	☐	For Bayesian analysis, information on the choice of priors and Markov chain Monte Carlo settings
☒	☐	For hierarchical and complex designs, identification of the appropriate level for tests and full reporting of outcomes
☐	☒	Estimates of effect sizes (e.g. Cohen's *d*, Pearson's *r*), indicating how they were calculated

Our web collection on statistics for biologists contains articles on many of the points above.

Software and code

Policy information about availability of computer code

Data collection	Microsoft Excel
Data analysis	Commercial software used include: Geneious v2022.2.0, Terra. Open source workflows used include: viral-ngs v2.1.33.16 (workflows: demux_only, classify_single, assemble_denovo, align_and_count_mulitple_report). All workflows are linked in the methods.<br><br>Other open source software used include: DIAMOND v2.0.15 , IQ-TREE v1.6.12, MAFFT v1.5.0, FigTree v1.4.4, qiime2 v2022.2.0, and ncbi-blast.<br><br>Custom scripts used include: script to retrieve least common ancestor of top blast/DIAMONd hits (lakras/bio-helper-415 scripts/blast/ retrieve_top_blast_hits_LCA_for_each_sequence.pl) and the Borrelia prediction models (github.com/colabobio/borrelia-diagnosis-prediction-models). These scripts are linked in the Code Availability section.

For manuscripts utilizing custom algorithms or software that are central to the research but not yet described in published literature, software must be made available to editors and reviewers. We strongly encourage code deposition in a community repository (e.g. GitHub). See the Nature Portfolio guidelines for submitting code & software for further information.

Data

Policy information about availability of data

All manuscripts must include a data availability statement. This statement should provide the following information, where applicable:

- Accession codes, unique identifiers, or web links for publicly available datasets
- A description of any restrictions on data availability
- For clinical datasets or third party data, please ensure that the statement adheres to our policy

-unbiased RNA-mNGS have been deposited in NCBI's SRA under BioProject BioProject PRJNA662334 (Accession numbers: SRR24622550-SRR24622641, SRR24995052-SRR24995258)
-16S demultiplexed sequencing reads have been deposited in SRA under BioProject PRJNA662334
-Complete and near complete (>80%) viral genomes assembled have been submitted to GenBank and accession numbers are available in Supplementary Table 1. These genomes have yet to be release by GenBank.

Research involving human participants, their data, or biological material

Policy information about studies with human participants or human data. See also policy information about sex, gender (identity/presentation), and sexual orientation and race, ethnicity and racism.

Reporting on sex and gender	In this study, "female" and "male" refer to patient reported sex. We did not collect information on gender identity. For samples obtained from Senegal, we collected approximately equal numbers of male and female subjects. Pregnancy and lactation were not exclusion criteria in this study. Contraception was not required to participate in this study. As we note in the text, there were more male febrile cases and more female controls, but the difference between groups was not statistically significant.
Reporting on race, ethnicity, or other socially relevant groupings	Since the subject enrollment takes place in West Africa, 100% of the study population is black, African. There were no exclusions based on race, ethnicity, or socio-economic status. The majority (66%) of the study population self-identified as Wolof, but other ethnicities were also represented.
Population characteristics	Participants raged in age from 2 to 75 years old and approximately half (49%) of participants were below the age of 18.
Recruitment	We performed a cross sectional study of febrile and healthy individuals. Febrile cases were selected from patients presenting to the SLAP outpatient clinic in Thiès, Senegal during the collection period under local IRB (SEN15/46) and Harvard IRB (IRB19-0023). During times when the study personnel were onsite, all patients who met the following inclusion criteria and gave consent were enrolled: (1) Febrile symptoms within the 3 days up to and including the day of presentation, (2) Age 2-75 years, and (3) Ambulatory with no signs of severe malarial (glucose < 2.2mM, hemoglobin < 5 gms/dL). Healthy controls were recruited via a call for participants. All volunteers who met the following inclusion criteria and consented were enrolled: (1) No febrile symptoms within the 3 days up to and including the day of presentation and (2) Age 2-75 years. In each season, enrollment continued until approximately the desired number of participants was reached. In 2018, we aimed for 200 febrile cases and 200 healthy controls in each season. In 2019, to avoid overrepresentation of well patients, we aimed for 100 febrile cases and 50 healthy controls in each season.<br><br>Informed consent was obtained for all enrollees (febrile and healthy); for minors under 18 years of age or individuals unable to provide their own consent, the consent of a parent or legal guardian was obtained. The study team explained to potential participants that their participation was strictly voluntary and that they could withdraw from the study at any time without any penalties or consequences and translations were made for potential participants (or their parent / legal guardian) that do not understand or cannot read the language in which the consent form was produced.
Ethics oversight	Samples were collected under local IRB approval (SEN15/46) and Harvard IRB (IRB19-0023)<br><br>This study was designed, implemented, and analyzed in close collaboration between US-based researchers and Senegalese researchers at Cheikh Anta Diop University/CIGASS, the SLAP outpatient clinic, and the National Malaria Control Programme. All study team members are included as authors and their contributions are detailed in the CRediT statement. The protocol was approved by the Harvard IRB as well as the IRB of the Ministry of Health in Senegal and all co-authors who worked directly with samples in Senegal or the U.S. received appropriate biosafety training. Sequencing and analysis was conducted at the Broad Institute due to higher capacity for high-depth sequencing. Sample aliquots are stored at both CIGASS and the Broad Institute, and all sequencing data and sample-associated data is accessible to study teams at both locations. Relevant research from other local teams is cited as appropriate.

Note that full information on the approval of the study protocol must also be provided in the manuscript.

Field-specific reporting

Please select the one below that is the best fit for your research. If you are not sure, read the appropriate sections before making your selection.

☒ Life sciences ☐ Behavioural & social sciences ☐ Ecological, evolutionary & environmental sciences

For a reference copy of the document with all sections, see nature.com/documents/nr-reporting-summary-flat.pdf

Life sciences study design

All studies must disclose on these points even when the disclosure is negative.

Sample size	In 2018, we aimed to enrolled 200 cases and 200 controls in each season (rainy, dry). In 2019, we aimed to collect 100 cases and 50 controls in each season. These sample sizes were primarily determine by collecting the maximum number of samples possible in order to identify rare pathogens that could be reasonably collected and processed with the available funds.
Data exclusions	When analyzing the continuous clinical variables (including blood glucose, heart rate, temperature, and hemoglobin) data, the data was checked for extreme outliers (z score > 10). One extreme outlier was found, a heart rate recorded at 389 beats per minute, which is non-physiologic and likely a result of inaccurate data input. This value was set to n/a.
Replication	All qPCR assay were performed in technical triplicate and the average Ct was reported. A sample was considered positive for a givne assay only if 3/3 replicates amplified.<br><br>Chemokine and cytokine measurements were performed in technical duplicates and the average value is reported.
Randomization	In this study, the febrile group was determined by who presented to the clinic with febrile illness during the collection period. Any patient who presented to the clinic during the times the study personnel were on site was assessed to determine whether they met inclusion criteria. If the patient met inclusion criteria and consented, they were enrolled. Enrollment continued in this matter until the desired number of participants for each season had been met.<br><br>Healthy controls were recruited via a call for participants after the completion of enrollment for febrile patients. In order to account for age as a potential co-variate, we aimed to enroll approximately equal numbers of controls as cases in each age group.
Blinding	Blinding was not possible in this study, given that participant groups were determined by their symptoms and not assigned.

Reporting for specific materials, systems and methods

We require information from authors about some types of materials, experimental systems and methods used in many studies. Here, indicate whether each material, system or method listed is relevant to your study. If you are not sure if a list item applies to your research, read the appropriate section before selecting a response.

Materials & experimental systems

n/a	Involved in the study	
☒	☐	Antibodies
☒	☐	Eukaryotic cell lines
☒	☐	Palaeontology and archaeology
☒	☐	Animals and other organisms
☒	☐	Clinical data
☒	☐	Dual use research of concern
☒	☐	Plants

Methods

n/a	Involved in the study	
☒	☐	ChIP-seq
☒	☐	Flow cytometry
☒	☐	MRI-based neuroimaging
